# Cancer patients as parents: implementation of a cross sector service for families with adolescent and young adult children

**DOI:** 10.1186/s12913-023-09413-8

**Published:** 2023-05-10

**Authors:** X. Skrabal Ross, F.E.J. McDonald, S. Konings, E. Schiena, J. Phipps-Nelson, F. Hodgson, P. Patterson

**Affiliations:** 1 Policy and Patient Department, Canteen Australia, GPO Box 3821, Sydney, NSW 2001 Australia; 2 Faculty of Medicine and Health, The University of Sydney, Sydney, Australia; 3 Psycho-Oncology Department, Clinique Saint-Jean, Brussels, Belgium; 4 Department of Allied Health, Peter MacCallum Cancer Centre, Melbourne, Australia; 5 Department of Oncology, University of Melbourne, Melbourne, Australia; 6 Office of Cancer Research, Peter MacCallum Cancer Centre, Melbourne, Australia; 7 John Hunter Hospital, New Lambton Heights, Australia

**Keywords:** Oncology, Parental Cancer, Adolescents, Young adults, Family support, Health Systems

## Abstract

**Background:**

Cancer patients who are parents show concerns about their ability to parent following diagnosis, and their adolescent and young adult (AYA) children have a need for improved cancer communication within the family. However, psychosocial support for families affected by parental cancer is not routinely available. This study explores the implementation of the Parent Support Worker (PSW) role, as part of a new cross sector model of care to support parent patients, their partners, and AYA children.

**Methods:**

Two PSWs, social workers and healthcare staff (n = 26) from three hospitals participated in audio-recorded, semi-structured interviews about implementation of the PSW role. Template Analysis and Normalization Process Theory were used to analyze the interviews. Data on PSW service activity and referrals of AYA to support from a community organization were analyzed using descriptive statistics.

**Results:**

Eleven themes categorized into enablers and barriers of implementation were identified. Regarding acceptability of the role, three enablers (social workers’ understanding of the PSW role increasing, easy and prompt access of staff and parent patients to PSWs, satisfaction with the PSW role) and one barrier (communication related confusion and frustration about the PSW role) were identified. Additionally, three enablers (the PSW role fills gaps in parenting-focused support and continuity of care, the PSW role alleviates social workers’ workload, negotiation helped to define responsibilities) and one barrier (fear of social work roles to be overtaken by PSWs) for appropriateness of the role were found. Finally, two enablers of feasibility of the role (PSWs and social workers co-managing the work, higher confidence from hospital staff to talk about children in the family) and one barrier (lack of systematic identification and referral processes) were identified. Across hospitals, the number of referrals of AYA children to the community organization increased between 2.7 and 12 times nine months post-introduction of the service.

**Conclusions:**

Established in response to identified gaps in oncology care for parents with cancer, their partners and AYA children, a novel cross-sector model of care was acceptable, appropriate, and feasible. Barriers and enablers to implementation identified in this study need to be considered when designing and implementing similar services.

**Supplementary Information:**

The online version contains supplementary material available at 10.1186/s12913-023-09413-8.

## Introduction

It is estimated that approximately one quarter of the adults diagnosed with cancer worldwide are parents of children, adolescents or young adults [1]. In Australia, more than 21,000 adolescents and young adults (AYA; 12–24 y/o) have a parent diagnosed with cancer every year [2–5]. Cancer disrupts normal family life, routines, and roles [6, 7]; partners face more responsibilities; and children have to deal with missing important activities and being cared for by different people in the absence of their parents [8]. After a cancer diagnosis, confidence in parenting abilities tends to decrease due to concerns about the impact of parents’ mood and physical changes, changes in family routines, and the emotional distress the diagnosis can cause on their children [9]. Unfortunately, family members of adult cancer patients tend to miss out on psychosocial support [10–12], including children [13–15].

The last decade has seen the development of a body of research highlighting the significant psychosocial burdens experienced by children of cancer patients, particularly AYA children. Previous reports show that over 60% of these AYAs have clinically elevated levels of psychological distress and high levels of unmet needs for information about their parents’ cancer, support from other young people, and open communication within the family about the cancer [16]. Due to their increased cognitive and abstract thought abilities, AYA are more aware about the potential consequences of cancer than younger children. In addition to this, a parental cancer experience poses challenges to AYA’s needs for independence and autonomy which makes them more susceptible to distress than younger children [17, 18].

For parents, communicating competently with their children about cancer is one of their greatest concerns, and many express a need for professional support in this area [7]. Challenges to address parents’ needs are global as this support is not embeded in standard practices [19] and oncology medical specialists often do not feel adequately equipped to discuss parenting issues, especially when the disease is in advanced stages. Barriers to discussing parenting issues include lack of time, confidence, knowledge, or emotional challenges [20–22]. Further, oncology nurses have expressed a need for peer-support and guidance on how to communicate with adolescents who face parental cancer and to assess the needs of these families as they feel ill-equipped to do so [23, 24]. Interventions addressing family functioning (a strong predictor of positive outcomes for AYA) [25] and effective communication (a component of family functioning) about the cancer diagnosis can alleviate families’ distress and improve parenting outcomes [19, 26–28], but have not been routinely implemented in healthcare [8, 19].

There is a recent increase in efforts to provide patients with integrated care that addresses patients’ holistic needs, including family issues. The “Accountable Health Communities” model in the United States and The NSW Integrated Care Strategy in Australia are examples of recent efforts to improve patients’ access to community-based support with strong focus on communication and coordination of care between hospitals, health professionals and community services [29, 30]. The greatest potential to improve the effectiveness and quality of healthcare involves the coordination and integration of the health sector and community-based organizations, as this linkage can help to fill the healthcare gaps that are essential to meet the broad needs of patients [31]. This paper reports on the implementation of an Australian initiative aiming to improve the provision and integration of care to families with AYA children impacted by parental cancer, by placing dedicated support workers within hospitals.

### The parent support worker role

The Parent Support Worker initiative is a novel cross-sector model of care which placed dedicated social workers called parent support workers (PSWs) within adult oncology wards, where they worked in collaboration with hospital social work teams to identify and provide support to parent patients with AYA children. The initiative was developed in line with strategies to provide integrated healthcare, and following the framework for development of models of care from the New South Wales Agency for Clinical Innovation [32].

The main function of the PSW role was to provide direct specialist support to parents with cancer (and their partners) including counselling, psycho-education, and practical information related to cancer, the impact of cancer on family and on parenting roles, and how to talk about cancer in the family. Additionally, PSWs provided secondary consultation to colleagues within the hospital social work department and other hospital departments regarding the support of parents with cancer, and ensured that young people (12–25 years) in the family were offered support through face to face and online services from a community-based organization^1^. While all PSWs were qualified social workers, no additional training was undertaken for this role.

To date, philanthropic funding and hospital support allowed piloting of the new model of care by placing three PSWs in three hospitals. Due to the importance of adapting the PSW role to the specific hospital contexts and to satisfy funder requirements, the implementation and characteristics of the role differed between the three institutions. Two PSWs worked part-time each in one hospital (3 and 4 days per week) and were hospital employees with roles funded via external grants, while one PSW assigned to another hospital worked full-time and was funded and managed by the community-based organization. Interaction between PSWs was minimal, with each PSW working independently and in line with the internal policies and procedures from the hospitals they were affiliated with. However, PSWs provided monthly service activity reports to the community-based organization, and liaised with staff from this organisation when referring AYA children for support. Prior to the initiation of the PSW service, one hospital consulted with their key staff and planned changes for the social work teams to make room to the new service. These changes included communicating the aims and duties of the PSW role to hospital social workers, revising the team’s processes for patient referrals between social workers, establishing the criteria for decisions about case management, and setting expectations about communication between social workers and PSWs when cases are co-managed. Due to time constraints associated with external funding of the PSW roles, the other two hospitals were unable to introduce measures to facilitate the introduction of the PSW role until after the PSW had begun working. As the PSW role represents a novel model of care for families impacted by parental cancer, evaluation work was undertaken to assess the implementation process, including barriers and facilitators to the introduction of the role, as well as their impact on care provision.

### Current study

This study aimed to explore the implementation of the PSW role in three Australian hospitals; specifically, the acceptability, the appropriateness, and the feasibility of the role based on Proctor et al.’s implementation framework [33]. Barriers and enablers were explored for each of these implementation outcomes. As part of the implementation process, service activity data on the PSW role were collected, with the aim to descriptively analyze and present data on the number of families supported by the service, their demographic characteristics, and the number of secondary consultations provided to hospital social workers and clinical staff. A study evaluating the impact of the PSW service on parents with cancer and their partners is underway, and will help to complement findings from this study by understanding families’ experiences of receiving care from PSWs.

## Methods

### Study design

A mixed-methods approach was undertaken to explore the implementation of the PSW role. Qualitative data was gathered through one-on-one semi-structured interviews with staff from the three hospitals, including the PSWs. Analysis of this data allowed exploration of the acceptability, appropriateness, and feasibility of the PSW role (based on Proctor et al.’s implementation framework) [33], and enablers and barriers to these implementation outcomes (based on Normalization Process Theory; NPT) [34]. The interviews were conducted by a Research Officer and SK, both with Psychology qualifications and experienced in conducting interviews for psycho-oncology research. The interviewers did not have a previous relationship with the interviewees. Qualitative data were collected until saturation was reached, that is, when no new information is discovered from data analysis. Quantitative data collected to monitor service activity and data on referrals of AYA to support from a community organization before and during the implementation of the PSW role (collected by the community organization) were analyzed to describe service activity and to explore the feasibility of the PSW role.

### Participants

Two PSWs and twenty-four staff members from the three hospitals (described as H1, H2, and H3) participated in interviews to explore their experiences with the implementation of the role (H1 = 7, H2 = 9, H3 = 8; 88% female; 14 social workers, 5 nurses, 3 managers, 1 psychologist and 1 oncologist).

### Procedures

A convenience sampling approach was employed to recruit participants for the interviews. Hospital staff received a printed information brochure with an invitation to participate in the study. PSWs were contacted directly via email with this information. Consent was considered implicit from participants’ self-nomination to participate, and participation on the interviews. The interviews were audio-recorded (average duration 41.9 min), transcribed verbatim and checked for accuracy against recordings. These took place 7 to 11 months after the introduction of the PSW role in the hospitals and were conducted in a hospital private room or via telephone.

### Data Collection

#### Quantitative data

The community organization collected monthly service activity data from the PSWs. Data included the number of referrals, clients, sessions, as well as clients’ demographics, information on family members attending the sessions, secondary consultations to hospital social workers and clinical staff and other relevant information.

Data on referrals of AYA children made by hospitals (routinely collected by the community organization) was gathered to extract the number of referrals made from the three hospitals before and following the implementation of the PSW role.

#### Qualitative data

Template Analysis, an approach to qualitative analysis (and form of Thematic Analysis) that utilizes highly structured analysis processes (e.g. development of coding template applied to data)[35] was used to inform the interview template and data analysis. This approach supported the preparation of an initial coding template using broadly defined *a priori* theme headings based on implementation outcomes (Table 1) from Proctor et al.’s implementation framework [33]. The three implementation outcomes that are most commonly used to evaluate the implementation of health interventions [36] were used in this study:

##### Acceptability

The perception among hospital staff that the PSW role is agreeable or satisfactory.

##### Appropriateness

The PSW role is perceived by hospital staff as relevant or compatible with their practice. It is also perceived as addressing an issue or problem within the practice.

##### Feasibility

The extent to which the PSW role can be successfully carried out or used within oncology settings and facilitates the referrals of AYA children to support from a community organization.

In this study, the NPT framework [34] also informed the design of the interview template and was used as a framework for data analysis. NPT is a sociology theory developed to explain how new processes become normalized within a healthcare context. It is concerned with the social organization of the work around the intervention (implementation), the practices that become a routine (embedding), and the sustainability of embedded practices into social contexts (integration) [37]. It has been used in multiple studies to design data collection tools, evaluate barriers and enablers of integration of new interventions into health contexts [38, 39], and make clear recommendations for improvement of processes [40]. NPT proposes that the integration of a new process requires work from the entire team and can be best understood by examining what staff members think and do in relation to the new intervention. NPT consists of four constructs that represent implementation work related to the intervention: a) coherence (how people make sense of the intervention), b) cognitive participation (how people ”intellectually” buy into the intervention), c) collective action (how people engage with the intervention), and d) reflexive monitoring (how people understand the intervention) [34].

As expected with Template Analysis, in this study each participant’s responses added depth and altered the structure of the coding template, resulting in additional themes to the ones set *a priori*.

**Table 1:** Interview Topics Based on Normalization Process Theory. (adapted from May and Finch, 2009 [34]

Normalization Process Theory Constructs	Definition	Implementation Outcome	Interview Topic	Examples of Interview Questions
Coherence	How interviewees “make sense” of the intervention	Acceptability of the PSW role	Whether staff demonstrate clear understanding of the purpose and differentiation of the service	What is the purpose of the PSW role? How does it differ from other established roles?
		Appropriateness of the PSW role	Whether staff perceive the role is needed in oncology practice and social work teams	Is there a need for the PSW role?
Cognitive Participation	How interviewees buy into the intervention	Acceptability of the PSW role	Feelings towards the role, and levels of buy in from staff	How do staff feel about the PSW role?
		Appropriateness of the PSW role	Whether staff consider the role is useful to oncology and social work practice	Is the PSW role useful for the team and the service?
		Feasibility of the PSW role	Barriers to access PSW service	What are the challenges to PSW service provision?
Collective Action	How interviewees engage with the intervention	Appropriateness of the PSW role	Impact of the role on social work processes	How did the social work team make room for the PSW role?
		Feasibility of the PSW Role	Collaboration between social workers and PSWs	How do social workers and PSWs work together? Do staff and social workers consult with the PSW?
			Whether staff access and refer to PSWs	Do staff routinely ask whether patients are parents? Do staff refer parent patients to the PSW?
Reflexive Monitoring	How interviewees understand the intervention	Acceptability of the PSW role	Understanding of the role over time	How did staff’s understanding of the role change over time?
		Appropriateness of the PSW role	Advantages and disadvantages of the role to oncology and social work practice	What are the advantages and disadvantages of the role to oncology and social work?
		Feasibility of the PSW role	Improvements in service delivery	Do staff members feel more confident with their support skills?
			Observations around quality of recognition, support, and care for parent patients afforded by the service	Do staff member observe differences within the team and its support of parents as a result of having a PSW?

### Data Analysis

Using a Template Analysis approach, interviews were analyzed by SK and one researcher officer by iteratively coding statements into themes in NVivo 12 [41]. The coders discussed the identified themes when disagreements were present, until a 100% agreement was reached. A third researcher (XSR) confirmed agreement with the themes.

The final themes were further interpreted using the four NPT constructs (coherence, cognitive participation, collective action, and reflexive monitoring) to describe barriers and enablers of the PSW role implementation (acceptability, appropriateness, and feasibility).

Descriptive statistics were used to provide an overview on the service activity data and to compare the number of referrals of AYAs to support from a community organization corresponding before and during the implementation of the PSW roles.

### Ethical considerations

The study was conducted in accordance with the principles of the revised Declaration of Helsinki, a statement of ethical principles which directs physicians and other participants in medical research involving human subjects. Prior to participation in the study all participants received written information about the research. Permission was also obtained to record the interview with an audio recorder. Participant informed consent was implied by their participation in the interviews, and this was approved by the Ethics Committees. This study received ethics approval from the Peter MacCallum Cancer Centre Human Research Ethics Committee in Victoria, Australia, ID: HREC/17/PMCC/210. Governance authorisation was obtained for the three hospitals where the study was conducted: Hunter New England Research Ethics & Governance Office ID SSA/18/HNE/122, South Western Sydney Local Health District ID SSA/18/LPOOL/259, and Peter MacCallum Cancer Centre Human Research Ethics Committee ID SSA/18/PMCC/12.

## Results

Results are presented in two parts. Part 1 provides the quantitative results including PSW service activity data and data on the number of AYA referrals to support from a community organization before and during the implementation of the PSW roles. Part 2 presents the common themes identified in the interviews using Template Analysis. These themes were categorized into implementation enablers and barriers of the PSW service according to NPT constructs.

### Part 1. Quantitative results

#### Service activity data

The first two PSW roles were established in October 2017 (H1 and H2), with the final role implemented in June 2018 (H3). The PSW service at H2 finished in October 2018 (H2), while the service is on-going in two hospitals (H1 and H3).

From October 2017 to September 2019 (24 months), PSWs supported 630 families through 1243 sessions across all hospitals. Parents were mostly female (65%), with average age of 46 years (range = 22–79 years). On average, 26 new families were supported by PSWs each month through 52 sessions across all hospitals. It is important to consider that from October 2017 to September 2019 the number of active PSW services varied. Although two PSW roles were active between October 2017 and September 2019, it was only between June and October 2018 that the service was available at all three hospitals.

Other hospital staff were supported through 142 secondary consultation sessions (average of 6 sessions per month) where they were provided with clinical advice and support to better assist parents. Table 2 presents the service activity data from the collaborating hospitals.

**Table 2:** Parent Support Worker Service Activity Data from the Three Hospitals

	New PSW Clients		PSW Client Sessions		Secondary Consultations	
	n	Monthly average	n	Monthly average	n	Monthly average
October to December 2017^a^	62	21	95	32	9	0.7
January to December 2018 ^b^	315	26	673	56	128	11
January to September 2019^c^	253	28	475	53	5	0.5
Total	630	26	1243	52	142	6

^a^ 2 active PSWs from October to December 2017, ^b^ 2 active PSWs from January to May and November to December 2018, 3 active PSWs from June to October 2018^, c^ 2 active PSWS from January to September 2019.

#### Referrals from hospitals to support from a community organization for AYA

The number of AYA referrals made to the community organization by the three hospitals increased notably (between 2.7 and 12 times) after the PSW role was implemented, compared to the nine months before the implementation of the role (see Table 3).

**Table 3:** Referrals from Hospitals to Support from a Community Organization for AYA Before and During the Implementation of PSW Roles

	Referrals to Community-Based Support (9 Months Prior to the Implementation of the PSW role)		Referrals to Community-Based Support (Last 9 Months During PSW role up to September 2019)	
Hospital	n	Monthly Average	n	Monthly Average
H1	15	1.6	41	4.5
H2	0	0	9	1
H3	3	0.3	36	4

### Part 2. Themes, enablers and barriers to implementation of the PSW Role

The results of the Template Analysis of the interviews are presented under three overarching topics (implementation outcomes) aligned with the coding template. The themes were also categorized into enablers and barriers to the implementation of the PSW role according to the implementation process and NPT construct they were aligned with: acceptability of the PSW role (4 themes; 3 enablers and 1 barrier), appropriateness of the PSW role (4 themes; 3 enablers and 1 barrier), and feasibility of the PSW role (3 themes; 2 enablers and 1 barrier). Example quotes are provided under the themes and additional example quotes are offered in Additional File 1. Some themes were found to be related to more than one NPT construct. Table 4 shows the categorization of the themes as enablers and barriers of implementation of the PSW role.

**Table 4:** Categorization of Themes as Enablers and Barriers of Implementation of the PSW Role

Implementation Outcome (topics)	NPT Constructs ^1^	Enablers (themes)	Barriers (themes)
Acceptability of the PSW role	Coherence	2.1. Social workers’ understanding of the PSW role increased with time	1.1. Lack of communication led to confusion and frustration about the PSW role
	Cognitive Participation	2.2. Satisfaction with the PSW role as it helps to increase support to families 2.3. Easy and prompt access (of staff and parent patients) to PSWs 2.1. Social workers’ understanding of the PSW role increased with time	1.1. Lack of communication led to confusion and frustration about the PSW role
	Reflexive Monitoring	2.1. Social workers’ understanding of the PSW role increased with time 2.2. Satisfaction with the PSW role as it helps to increase support to families	
Appropriateness of the PSW role	Coherence	4.2. The PSW role fills gaps in parenting specialized support and continuity of care	3.1. Initial fear for social work roles to be overtaken by PSWs
	Cognitive Participation	4.3. The PSW role alleviates social workers’ workload	3.1. Initial fear for social work roles to be overtaken by PSWs
	Collective Action	4.1. After some time, negotiation helped to define responsibilities	
	Reflexive Monitoring	4.2. The PSW role fills gaps in parenting specialized support and continuity of care 4.3. The PSW role alleviates social workers’ workload	
Feasibility of the PSW role	Cognitive Participation		5.1. Lack of systematic processes to identify parent patients in hospitals and refer them to PSWs
	Collective Action	6.1. PSWs and social workers co-managing the work 6.2. Higher confidence from hospital staff to talk about children in the family leads to more referrals to the service	5.1. Lack of systematic processes to identify parent patients in hospitals and refer them to PSWs
	Reflexive Monitoring	6.2. Higher confidence from hospital staff to talk about children in the family leads to more referrals to the service	

^1^ Definition of the NPT constructs. Coherence: How interviewees “make sense” of the intervention, Cognitive Participation: How interviewees buy into the intervention, Collective Action: Are interviewees engaged in the intervention? Reflexive Monitoring: What do interviewees think about the intervention?

### Acceptability of the parent support worker role

Early in the implementation of the PSW role, hospital social workers were confused about the PSW role due to a lack of communication. However, these barriers were overcome with time with hospital staff expressing their satisfaction with the role and reporting that they valued the easy access to PSW services.

### Barriers to acceptability of the parent support worker role

#### Lack of communication led to confusion and frustration about the PSW role (coherence and cognitive participation)

The lack of communication about the aims of the role presented as an issue for hospital social workers in two of the hospitals. They expressed a need for more transparent, clear communication prior to the role being launched. They felt confused and frustrated about not having a clear idea of the type of clients the PSW was able to service (e.g. children or parents), their role, and the expectations and limitations of the role, for example, when the PSW was not a health employee (due to funding arrangements) and was not able to provide the same support as other hospital social workers (e.g., providing patient discharge plans).

*“[PSW] basically started. Like, the week before she started we got told that okay, [PSW] is coming to our team, we need to train her, we need to organise everything for her and… we just don’t know what to do, where to start because I think a lack of communication, or lack of transparency from the staff, that make it harder for us to work through things” IDSW24.*

“*I think it’s taken a while for everyone to understand that actually, [PSW’s] not a health employee, she’s a [community organization] employee. I think that comes, sometimes that has created tension in terms of wanting her to do more health work, but she can’t*” *IDSW18*.

### Enablers of acceptability of the parent support worker role

#### Social workers’ understanding of the PSW role increased with time (coherence and reflexive monitoring)

At the time of the interviews (7–11 months after the implementation of the role), hospital social workers had a clear understanding of the PSW role. They expressed that PSWs supported parents and their children within AYA age group (12–25 years old), helped parents to communicate with their children about cancer, and supported children to cope with parental cancer. They understood this support could be provided across different hospital wards and the community. Additionally, PSWs were able to provide secondary consultations to other social workers about how to approach the parental aspects of cancer.

*“She is supporting the parents who have kids between 12 to 25, obviously the [AYA] category and it is just a complimentary to our team how she can actually support the parents who have cancer and also after the treatment in the community and in the hospital or in the ward if I need her, I can call her, too” IDSW24.*

#### Satisfaction with the PSW role (cognitive participation and reflexive monitoring)

Hospital staff were satisfied with the PSWs role, as they felt the team’s impact on care for patients (and their families) was higher than it was before the PSW role was introduced. Their satisfaction was also evident in staff desire for continuation of PSWs work in their hospitals.

*“It really impacts the way you feel about working with the family. That particular case gave me a lot of satisfaction in my work because I felt that we’d done a good job, we’d actually assisted this family through something difficult which doesn’t happen that often, unfortunately…you feel like you’re actually doing something. Having that extra hand on deck, I suppose” IDSW24.*

*“Well ideally, I’d like to see it continue because as far as I understood it was a pilot project for a year… my hope would be the ongoing support from [community organization] to be able to establish this as a regular role” ID23.*

#### Easy and prompt access (of staff and parent patients) to PSWs (cognitive participation)

Hospital staff valued their easy access to PSWs and their timely response to staff and patients’ service requests.

*“She’s only a phone call away. If she’s not available, one of the other social workers will take the message and they’ll pass it onto her as soon as she’s available. I don’t think she’s never not rung me back, she’s really, really good” ID22.*

### Appropriateness of the parent support worker role

At initial stages of the PSW role implementation, hospital social workers feared their roles being overtaken by PSWs. However, negotiation of responsibilities helped to overcome these barriers. The PSW role filled a gap in services to parent-patients and families, and helped to reduce the workload of hospital social workers.

### Barriers to appropriateness of the parent support worker role

#### Initial fear for social work roles to be overtaken by PSWs (coherence and cognitive participation)

Prior to the launch of the PSW role, some of the hospital social workers enjoyed and felt trained to work with patients (and family members) of all ages, including children. There was an initial fear from hospital social workers for their roles to be taken over by PSWs, which seemed to be a consequence of their uncertainty about the purpose of the new role.

*“I think initially within the social work team, there was probably some ... not ambiguity, uncertainty about the role and probably at the beginning it might have been seen as taking work away from some of my colleagues. Some of them particularly enjoy doing this work” (PSW1).*

### Enablers of appropriateness of the parent support worker role

#### After some time, negotiation helped to define responsibilities (collective action)

Later, after the introduction of the PSW role, overlapping responsibilities were negotiated to provide room for the new role. For example, initial negotiations took place to differentiate the work the social workers and PSWs did with children of different age groups, and ongoing negotiations took place when a hospital social worker (with no previous knowledge about the presence of children in the patient’s family) already formed a relationship with the patient. In these last cases a referral to PSWs did not take place. Instead, the social workers searched for validation and knowledge from secondary consultations with PSWs to enhance their skills in supporting parents and their children.

*“We can provide counselling to kids and that’s something that we always have passion to do… now after we work out a plan, we know the role a bit better, there’s no problem. I can still see kids, it’s just in a different setting and different age group. I see younger kids and [PSW] the 12 to 25 and sometimes I can still see within the [community organization] age group because it’s not appropriate for [PSW] to see. So, there’s no problem” IDSW24.*

#### The PSW role fills gaps in parenting specialized support and continuity of care (coherence, reflexive monitoring)

Hospital staff recognized the impact of cancer on parents and their children, and a pre-existing gap in the provision of specific parental support with emphasis on the family unit, which was filled by PSWs. The interviews highlighted that hospital staff may overlook providing support to children because they are not at the hospital with their parents, lack the knowledge about how to support children, or lack the time to provide family support. A pre-existing gap in continuity of care outside oncology wards and hospitals was also identified. PSWs covered this gap due to their capacity to continue supporting families outside these boundaries. Hospital staff services are limited by the hospital and ward they are affiliated with, while the PSWs did not face these restrictions. Additionally, PSWs in two hospitals were proactive in searching for parent patients in the hospitals, which is not a standard process in referral-based hospital social work.

*“The ward social workers can respond to any range of needs. This role is very much specific to the emotional needs of the parent going through cancer, and I guess also the ongoing support needs… It doesn’t have those competing 12 discharges this morning that all need my attention. There’s a lot more capacity for detailed support and concentrated support” PSW2.*

*“I like the role because she calls people before they come to hospital. And can continue to liaise and support them beyond the admission. So our role within the hospital is only when they’re an in-patient. So as soon as they’re discharged from hospital, we’re not really allowed to continue contact with the patient ... with the people” IDSW9*.

#### The PSW role alleviates social workers’ workload (cognitive participation and reflexive monitoring)

The PSW role helped to reduce the overloaded work schedules of hospital social workers and allowed them to focus on “doing their job” in supporting patients across other psychosocial needs. PSWs were considered to be a complement to hospital staff’s roles which strengthened the teams’ work by offering the parent-focused support delivered by PSWs.

*“I’ve greatly appreciated it and it has freed me up to be able to do my job, to be able to expand my service to see other people who I may have otherwise missed out because I was busy with children and different things” ID10*.

### Feasibility of the PSW role

The introduction of the PSWs in the hospitals led to changes in systematic parent identification processes and hospital staff’s confidence in talking about the presence of children in the family, which supported the viability of the role being carried out in the hospitals. The feasibility of the role was also supported by hospitals social workers’ utilization of the service and the co-management of patients with PSWs.

### Barriers to feasibility of the PSW Role

#### Lack of systematic processes to identify parent patients in hospitals and refer them to PSWs (collective action)

At the commencement of the pilot, hospital admission and outpatient assessment forms did not routinely include questions specifically asking about the presence of children in the family. Collecting this information relied on hospital staff’s education and willingness to do this. A “straight” referral to PSWs depended on the “chance” that clinical staff (nurses, treating specialists) asked if the patient had children, which was not a standard practice. Additionally, in two hospitals, there was no systematic referral pathway from clinical staff to PSW services. The lack of information about the presence of children in the family led to referrals being made to other social workers (due to other issues e.g. financial) who then had to refer the patient to the PSW, duplicating work. As a consequence of the introduction of the PSW role, two hospitals trialled a systematic assessment of the presence of children in the family (question at patient hospital intake).

*“There’s no formal checking of that [asking about children] in hospitals. That would be the doctor or nurse that should ever ask that. It should be asked at registration, I believe…staff that are taking that information are not necessarily trained in knowing where that information should go…there’s a lot of work being done here around, what’s called the [name of the electronic questionnaire]. And that has been piloted out across a couple of different clinical streams” PSW1*.

*“We are the social workers. If we feel like someone has children, then we refer to [PSW]. So there is double up at the start. It’s difficult” IDSW9.*

*“I don’t know how many of the specialists aren’t referring people who have children. I don’t know how many of the specialists are referring people who have cancer. They [the specialists] wouldn’t be asking whether they’ve got kids or not, it would only come up serendipitously during the conversation” IDSW8.*

### Enablers of feasibility of the PSW Role

#### PSWs and social workers co-managing the work (collective action)

Social workers valued the collaboration with PSWs in co-managing cases, especially those of higher complexity, and they consistently referred parents to this service or had secondary consultations with PSWs to ensure parents’ support needs were addressed.

*“I feel like it’s good, because then I can just focus on the patient and what they want to get out of the social work relationship. Then [PSW] can work on the other side… it’s really good, it’s almost like co-case managing in a way, but we work very yeah, together” IDSW21*.

*“It’s sort of just communicating to each other what our impressions and plan are so that that can be complementary and coordinated in any way, particularly if [PSW] might not necessarily see them in an ongoing manner but I am, how can I draw off what’s been communicated and ensure that that’s able to be confirmed or assist patients in accessing what they need down the track” IDSW3.*

#### Higher confidence from hospital staff to talk about children in the family leads to more referrals to the service (collective action and reflexive monitoring)

The PSW role increased hospital staff’s (clinical and social workers) confidence in having initial conversations with patients about their parenting and their children’s needs, which led to more referrals to the PSW service. These conversations were previously avoided by some of the staff due to their lack of confidence about the topic.

*“I think it [PSW role] just gives you a bit more confidence to have those initial discussions. In the past a lot of people, including myself at times, have been somewhat avoidant of these sort of conversations because they didn’t know how to do it or we thought we were going to mess things up or we lacked a bit of confidence in doing those things” ID8.*

## Discussion

This study explored the implementation of the PSW role in three Australian hospitals. Over two years, across all hospitals, the PSWs worked directly with over 600 families and supported other hospital staff through over 100 secondary consultation sessions to better assist families affected by parental cancer. Referrals of AYA children to support from a community organization were shown to be feasible through the PSW role with the number of referrals from the three hospitals increasing during the nine months following role implementation, translating into a notable increase in reach to specialized support by AYAs who are in a vulnerable situation during their parental cancer experience [16]. However, findings from qualitative interviews with PSWs and healthcare professionals highlight challenges that initially hindered the introduction and operation of the PSW role, as well as identifying efforts and factors which facilitated the integration of the role into standard care practices. Ultimately, the findings from this study show the PSW role to be highly accepted and appropriate to hospital social work and clinical staff, and feasible to be implemented into hospital oncology care, with appropriate forward- and ongoing planning.

Findings from research on this cross-sector model of care represent a contribution towards growing worldwide initiatives to provide cancer patients with integrated care through collaboration between health care institutions and community-based organizations [29, 42], and learnings from the implementation of the role may be instructive for other healthcare services seeking to improve care for families impacted by cancer.

Previous studies highlight that the specific psychological, communication and information needs of parents with cancer and their AYA children [16] are not routinely identified and addressed by standard oncology health services [8, 19]. Hospital staff’s workload and lack of time are the most common barriers to implementation of hospital-based health interventions in general [36] and interventions for parents with cancer [37]. As highlighted by interviews in this study, while the lack of systematic procedures to identify patients with children was a barrier of implementation of the role, the PSWs alleviated hospital social workers workload and provided a specialized service that hospital staff did not have the time or felt well-equipped to offer.

The introduction of the PSW role increased the capacity of healthcare teams, filling a gap in the provision of specialized support and continuity of care to parents with cancer and their families. This is evident in both the qualitative testimonies of interviewees about the acceptability and appropriateness of the PSW role, and in service data collected by the community organization, demonstrating an increase in the monthly average of new PSW clients, sessions and referrals year for year (despite fluctuating numbers of PSWs operational). The new model of care therefore expands hospitals’ capacities to provide holistic care to families impacted by parental cancer through increased integration of clinical and community support services. However, this was contingent on external funding of the PSW roles, which may not be sustainable over time- as was the case with one of the hospitals in this study, which did not have the funding to support the PSW role beyond this study. The implementation of future services similar to the one described in this study should adequately plan for sustainable ways to fund the roles over time.

The systematic assessment of the presence of children in the family of cancer patients in hospitals (including clear referral pathways to specialized support for families) and the use of available information resources for parent patients amongst hospital clinical staff may help to improve access of parents to intervention and support information. In this study, the lack of systematic referral pathways of parent patients to PSW services from hospital staff was found to be a barrier to the feasibility of the role in two of the hospitals. In these hospitals, although supported by social workers and some clinical staff, most of the patients in need of specialized parenting support were identified by the PSWs’ active search for parents. The other hospital counted with defined patient referral pathways to PSWs from hospital social workers or health professionals.

Clinical staff are the most promising referral source to parent specialized services, but this was a challenge in our study. Possible barriers for referrals from clinicians to services similar to the one described in this study include their lack of awareness about children in the family of adult oncology patients [15] and the time, emotional, confidence, and knowledge barriers experienced by oncology medical specialists and social workers to deliver parent-specific information [6, 7, 20, 21, 23, 26]. These barriers highlight the need for the PSW role and also emphasize that education for clinical staff about parenting challenges should be extensive and ongoing to increase referrals to the PSW service and better support families. Information resources have been developed to support parent patients, to improve communication between them and oncology medical specialists, and to raise clinical staff’s awareness of AYAs parenting-related issues (45). However, these resources are not consistently employed in clinical settings. Strategies to make this information widely available to and utilized by clinicians should be explored by future research.

The introduction of questions about parenting status at hospital admission or intake (with clear referral pathways to parenting services) is another strategy with potential to improve parent referrals to the specialized support. While this is not a standard practice in Australian hospitals [7] and in other countries, for example Germany [43], the implementation of the PSW role led to the trial of new questions about parenting status at hospital admission in two of the hospitals, due to combined PSWs and hospital staff efforts to improve referral systems to the service. Even in the absence of a dedicated PSW, introducing questions about patients’ families and children as part of routine screening and assessments may help to identify such families to their care teams, who may be able to provide more tailored support or referrals.

Hospitals, healthcare workers, and most importantly patients and their families, are best served where implementation of a new service is undertaken in consultation with staff and supported by an aligned communications strategy. This study highlighted that it is highly important to make changes to social work team processes and to communicate the aims of the PSW role to hospital social workers *before* the introduction of the role. While one hospital in this study followed this approach, in the other two hospitals social workers’ lack of information about the role prior to launch created confusion and fear of overlapping roles, creating barriers to the implementation and integration of the services. This is in line with previous evidence showing that knowledge and understanding of the aims of hospital-based health interventions (including those for parents with cancer) are key in their implementation and help to prevent staff confusion and resistance towards a new service (43, 44). In the future, the acceptability and appropriateness of new PSW roles could be facilitated by preparatory engagement with hospital social workers that help them to “make sense of” (coherence, NPT) and “buy into” (cognitive participation, NPT) the new role. This includes exploring whether they believe it is right to introduce the new role within their teams, encouraging ongoing opportunities to ask for clarification and providing feedback about the role, and including them in conversations about changes to important processes (e.g., changes in referrals, co-management of cases).

This study presents evidence about the implementation of an integrated and holistic cross-sector model of care that has important implications for health service delivery and policy making. The present research shows that the integration of parent-specific support as part of routine care has the potential to improve the quality of care in oncology settings and has a positive impact on hospital staff. This model of care is further hypothesised to support the health system in meeting the family needs in a streamlined, consistent, and coordinated way. The PSW model may also have value beyond oncology settings and could be applied to the broader context of chronic disease that pose similar challenges to cancer: namely protracted treatment protocols, side-effects, and social and economic impacts. Through this study, health policy makers are provided with an opportunity to consider a model of care which can be implemented across the health system for multiple patient cohorts. Findings from this study may serve to inform the development of similar cross-sector, specialized services for parents with cancer and their children, especially about barriers and strategies to enhance the acceptability, appropriateness, and feasibility of the service in hospitals. However, it is important to consider the individual characteristics and processes of each hospital (which are diverse even in the same country) when implementing these types of services.

### Study strengths and limitations

To our knowledge this is the first study to evaluate the implementation of a novel cross-sector model of care to provide in-hospital psychosocial support to parents with cancer, their partners, and AYA children. One of the methodological strengths of the study is the use of two implementation frameworks which allowed deeper analyses to identify barriers and enablers of implementation by considering the involvement of hospital staff in the process of implementation of the role. However, the use of the NPT model encompasses challenges, as understanding the model is time consuming and the process of coding can be complicated by the overlapping of themes into different constructs. These challenges have also been reported by previous studies [39].

The study included a range of healthcare professionals: social workers, nurses, managers, oncologists, and psychologists, ensuring multiple perspectives on implementation were represented. However, some findings of this study may be limited to healthcare professionals working in public hospitals in Australia, where the PSW role was implemented. These hospitals had management support of the role. Finally, the individual characteristics of the PSWs were not explored by this study. It is not clear how much the PSW’s skills, previous work experience, and personality played a role in enabling the implementation of the PSW role.

## Conclusions

This study explored the implementation of the PSW role as part of a novel cross sector model of care to support parents with cancer, their partners, and their children. The PSW role was found to be acceptable, appropriate, and feasible to be implemented into hospital oncology settings. It increased the number of referrals of AYA to support from one community organization and helped to build the capacity and quality of oncology care. Barriers and enablers to implementation identified in this study, for example the lack of communication about the PSW role and the value of the role in reducing the workload of hospital social workers, should be considered when designing and implementing similar services in the future.

Failure to adequately prepare for a new service like the one described in this study can lead to delays in effective assistance to parent patients and families at a crucial time. The PSW role has the potential to fill existing gaps and improve the continuity of healthcare to parent patients and their families across the health system by providing specialized psychosocial services that extend outside hospital settings. To increase the reach of parent-patients (and their families) to this support there is a need for hospitals to implement screening processes such as systematic parent identification. There is an opportunity for future health policy makers to consider the integration of parent-specific support as part of routine care, not only in oncology settings but also in the context of chronic diseases that may pose similar challenges to cancer. A study evaluating the impact of the PSW service on parents with cancer and their partners is underway and will help to complement findings from this study.

## Electronic supplementary material

Below is the link to the electronic supplementary material.

Supplementary Material 1

## Data Availability

The datasets generated and analyzed during the current study are not publicly available due to containing information that could compromise the privacy of research participants but are available from the corresponding author on reasonable request.

## Footnotes

1 Canteen Australia is an Australian non-for-profit organization that provides psychosocial and practical support to young people affected by a cancer experience (including their parents’ cancer) and their families.
