# Supplementary material for: Cancer patients as parents: implementation of a cross sector service for families with adolescent and young adult children
Source: BMC Health Serv Res. 2023 May 10;23:472. doi: 10.1186/s12913-023-09413-8 (PMC10173515; doi:10.1186/s12913-023-09413-8)
Supplement: Supplementary file 1 — Supplementary Material 1 [file 12913_2023_9413_MOESM1_ESM.docx]

**Additional File 1. Additional Sample Quotes**

| **Categories (implementation outcomes)** | **Themes** | **Sample Quotes** |
| --- | --- | --- |
| Acceptability of the PSW role (Barriers) | 1.1. Lack of communication led to confusion and frustration about the PSW role | *“But there was confusion …there was just no clear idea what her role was…The frustration is more around I don't know what she can or can't do. Like her limitations. What exactly is her role” ID24*  *“When they said we were getting it I’m going, “Why do we need a Canteen social worker?” Shouldn’t it be based out in Paeds, that’s where – you sort of hear Canteen and you go that’s for kids so shouldn’t they be based out in Paeds?” ID22*  *“I didn’t get much information at all before [PSW] started, but then when she did start, I took on a lot of that sort of role of orientating her to the hospital and to our team and to the clinical area and the position” ID19.* |
| Acceptability of the PSW role (Enablers) | 2.1. Social workers’ understanding of the PSW role increased with time | *“I think [PSW] is more specific about that, but the impact is, she's more aware of the impact on that family unit. We might still be aware of an impact on the family, but it's more about our patient and how we can discharge them safely” ID15.*  *“So looking at providing that ongoing counselling support to the parents and providing sometimes like advice and guidance on how to approach talking to children about cancer” ID20.* |
|  | 2.2. Satisfaction with the PSW role | *“Yeah - and just for us to know that [PSW] it’s there, it makes me much happier about this area. I wish we had more in other areas [laughs] to be honest. [There aren’t] support workers but oh well maybe one day [laughs]” ID18.*  *“I think it's been a great initiative. I think we're very lucky to have [community organization] on board and funding this. I want to be still continuing” ID2.*  *“I think it's been effective in a sense that parents feel like the health service they're attending is actually acknowledging the unique challenges they face” PSW1.* |
|  | 2.3. Easy and prompt access (of staff and parent patients) to PSWs | *“She was on to it straight away that day, she went out to the hospital and gave brochures to the family and provided support. That immediacy was really great, (a) because it just helped me out, but (b) it just gave the service to the family that were wanting it straight away” ID21.*  *“[PSW] was very good at picking up the patients the day after she gets back, so that's not a problem. And, if there is anyone by any chance that gets missed, it's just a matter of me phoning up and saying, "Oh, yeah. Do you mind touching base with this person?" So, she's very flexible in that sense” ID12.* |
| Appropriateness of the PSW role (Barriers) | 3.1. Initial fear for social work roles to be overtaken by PSWs | *“I understand that there was some like a bit of ambivalent – ambivalence… with some other like… areas, of the hospital…because it might be seen as like taking over the social work role that already exists” ID17.*  *“I can imagine that for some people that are um - yeah, less effective at communicating with workers those might get confused and I think maybe without the right person in the role, I have had a really good experience with [PSW], without the right person in the role you do run the risk of - I guess, overlapping with each other as well and some confusion about whose role is what” ID18.* |
| Appropriateness of the PSW role (Enablers) | 4.1. After some time, negotiation helped to define responsibilities | *“It just depends with - if I have a real established rapport with the family, um, then I will - it's unlikely that I'll refer to [PSW]… What I will do is I'll ask her if I can have, um, some of her resources from [community organization]… And I'll talk to her about what would be appropriate and then that she will provide me with the education… and I'll do it myself” ID17.*  *“Since [PSW’s] been on board, probably younger children has been more of my work load, so kids who don’t fit the [community organization] age group, yeah, and if I’m working with a family and they do have kids and then later down the track, they raise something with me around support for the kids, then I would get [PSW] to give them a call” ID19.* |
|  | 4.2. The PSW role fills gaps in parenting specialized support and continuity of care | *“They [hospital staff] don’t know how to talk to the kids, I don’t know how to talk to the kids…So now that [PSW’s] sort of based here it’s good because I know I can get her here” ID22.*  *“Here with [PSW] being a bit more proactive and finding out about those patients that are in the hospital…they're actually targeted to receive some of that support” ID15.*  *“I think there was definitely a need and I think sometimes there is that gap, when the patient leaves the hospital, around the ongoing support that they are going to be getting in the community and I think that that role helps fill that gap” ID20.*  *“I know [PSW's] very good at ringing up and phoning the patients a week, two weeks down the track once they have been discharged from home. And, I think that's really important that that continues. And, I ... You don't really get that with you mainstream social workers” ID12.* |
|  | 4.3. The PSW role alleviates social workers’ workload | *“I mean the one change that I would mention from that was really it was a reduction in my workload. It was because I knew that these issues could be discussed at length in a particular time in a proper therapeutic counselling session with [PSW]” ID18.*  *“She would give us more time to focus on the important things because we know that [PSW] can look after not only the kids, but she can provide very important information to the patients, how to support the kids and where I can actually focus on other practical things and the patient's emotional side. So, that gives us more time” CCH8.*  *“So, it did help me in time management side of things, 'cause rather than me having to go over things with them, [PSW] was able to talk about things” JSI05.* |
| Feasibility of the PSW role (Barriers) | 5.1. Lack of systematic processes to identify parent patients in hospitals and refer them to PSWs | *“I think it's something that ideally we would like to have screened at admissions or at registration because it's often we won't be made aware of it until someone's on the ward or if just they happen mention something in passing. It's not necessarily something that's regularly asked by the treating teams…I would prefer to know of it when they're an outpatient so that we can put supports in place earlier” ID5.*  *“There's nothing on our system that will identify that or have easily pulled that out. It relies on other staff picking that up, assessing that, or the patient, family themselves requesting social work…as social workers, we're aware” ID14.*  *A lot of it is ... we are the social workers. If we feel like someone has children, then we refer to [PSW] to that person. So there is double up at the start. It's difficult to ... yeah” ID9.* |
| Feasibility of the PSW role (Enablers) | 6.1. PSWs and social workers co-managing the work | *“If they have complex different issues like not only how to communicate to the kids, but with emotional sides, or practical things like housing issues, the partner needs some support too, I just think that one person can't manage so much, that's when I can ask [PSW] to come along and provide that help. So, just to divvy the work and we can provide the best we can for the patient and the family” ID24.*  *“Someone like myself might do an initial assessment and work with the …the parents on different things. Whereas then [PSW] might come into work on like more, um, linking the children up to, um, [community organization] services, um and supporting, like, sometimes the cases are quite complex, that you required two workers to kind of work with the family” ID17.*  *“The referral came to me when I was about to go on leave, so I asked [PSW] if she could make a start on it and she’s built this amazing relationship with his wife and we’ve been able to work together on it” ID19.* |
|  | 6.2. Higher confidence from hospital staff to talk about children in the family leads to more referrals to the service | *“Having some confidence in knowing that someone who knows what they're doing is going to be able to support and talk to the parent and the children together, about what's happening…I think I'm not the only one in the hospital that would worry about that and when in hospitals people worry about something they often avoid it” ID8.*  *“I was always sometimes standoffish about asking questions because I sometimes think if they ask me something difficult how am I going to answer it. But having the role of the [community organization] person here it sort of helped build a relationship and I know that I feel a lot more confident now being able to answer the questions that I can and then I say, “If I can’t help you with this I can get the social worker to come up” ID22.* |
